# Development and In Situ Application of Deformation Monitoring System for Concrete-Face Rockfill Dam Using Fiber Optic Gyroscope

**DOI:** 10.3390/s20010108

**Published:** 2019-12-23

**Authors:** Cheng Liao, Desuo Cai, Hongxun Chen, Weili Luo, Miao Li

**Affiliations:** 1 School of Civil Engineering, Guangzhou University, Guangzhou 510006, China; 1111816004@e.gzhu.edu.cn (C.L.);; 2 College of Hydraulic & Environmental Engineering, China Three Gorges University, Yichang 443002, China; caidesuo@vip.163.com; 3 Jiangxi Water Resources Institute, Jiangxi 330013, China

**Keywords:** Concrete-face rockfill dam, face slab deflection, FOG, deformation monitoring

## Abstract

Deformation monitoring is of importance to ensure the operation status of concrete-face rockfill dams (CFRD). This paper reported a novel fiber optic gyroscope (FOG) monitoring system for continuously monitoring face slab deformation of CFRD, which consisted of a permanent monitoring pipeline and a sensing vehicle. The monitoring pipeline was made of steel pipes and polyvinyl chloride polymer connectors, which was embedded in a slot of the crushing-type sidewall beneath the concrete face slab of CFRD, forming a permanent monitoring channel. The sensing vehicle was equipped with a high-precision FOG sensor. A low-pass filter was designed to eliminate the vibration noise of the angular velocities of the sensing vehicle during the monitoring process. An in situ test was carried out on the Shuibuya dam, the highest CFRD in the world. The measurements of the FOG monitoring system agreed well with traditional instrument measurements, serving as validation of the system. The FOG monitoring system has the advantages of excellent repeatability, long service life, distributed monitoring, and automatic measurement.

## 1. Introduction

Concrete face rockfill dams (CFRDs) are frequently constructed worldwide because of their cost-effectiveness, short construction periods, excellent adaptability to topography, inherent resistance to earthquake loading, and use of local materials [1,2,3,4,5]. For example, CFRDs are almost the only option for harvesting abundant hydropower resources in western China, which has a harsh natural environment and inconvenient transportation, a shortage of impermeable materials, and is an earthquake-prone area [6]. Nowadays, more than 600 CFRDs around the world have been constructed, are under construction, or are planned [7]. Table 1 lists representative CFRDs higher than 200 m [6,8].

One of the key issues of these high CFRDs is their safety during operation. If any of the dams collapse, it would pose a great threat to people’s lives and property downstream. It has been reported that some high CFRDs, such as Campos Novos dam (202 m, Brazil), Barra Grande dam (185 m, Brazil), Mohale dam (145 m, South Africa), Aguamilpa dam (187 m, Mexico), and Tianshengqiao dam (178 m, China), experienced significant structural breaks leading to considerable leakage, which affected their normal operation due to large-scale deformation of concrete face slab [9]. Hence, it is of importance to monitor the deformation dams, including the settlement and face slab deflection. To this end, many traditional instruments, such as tension wire alignment, hydraulic overflow settlement gauges, GPS, and inclinometers, have been adopted in the literature. For example, Dai et al. [10] reported the application of tension wire alignments to measure the internal horizontal displacement of a dam and to assess its operation. Zhou et al. [11] employed a hydraulic overflow settlement gauge to measure the inner settlement of the Shuibuya CFRD dam. Xu et al. [12] presented the application of a GPS external automatic monitoring system in the Geheyan Dam. Wen et al. [13] used inclinometers to measure the face slab deflection of the Miaojiao CFRD. More recently, the advanced synthetic aperture radar (SAR), which is accurate to a few millimeters and has high spatial resolution, was employed to monitor the surface displacement at the Pacoima dam [14], the surface deformation of the Genzano Di Lucania dam, and the settlement process of the Gongming dam [15,16]. Another high spatial-resolution technique known as the terrestrial laser scanning has also been developed for deformation change detection on the Las Cogotas dam [17] and deformation monitoring of the Changheba dam [18].

However, traditional instruments are a of the point-measurement type, and can only measure discrete points with a separation distance of 20~50 m. The installation of these instruments greatly affects the dam’s construction; conversely, the latter would damage the instruments or reduce their service life. In sum, traditional instruments cannot meet the long-term safety monitoring needs of CFRDs in terms of efficiency and accuracy, because of their low coverage efficiency, lack of durability, short service lifetime, and labor-intensive monitoring requirements. More advanced technologies, such as the SAR and terrestrial laser scanning, can only be used to monitor the external or surface deformation of CFRDs.

In this paper, a novel monitoring system is designed to monitor the face slab deflection of CFRD based on the original design concept of the authors’ team [19]. The advanced monitoring system mainly consists of a monitoring pipeline and a sensing vehicle equipped with a high precision FOG sensor. In comparison with the traditional instruments, the FOG monitoring system has three advantages: (a) the monitoring pipeline is installed with little interference to dam construction, forming a permanent monitoring channel which can provide long-term and effective monitoring for the safe operation of the dam, (b) the FOG monitoring system is a distributed measurement whereby the deformation of the whole section of the face slab can be obtained, and (c) the system is automatic and requires much less-intensive manpower. The working principle of the FOG monitoring system is described in detail. Finally, an in situ test on the Shuibuya dam is reported, and the results are presented in terms of the face slab deflection at different levels of the water table and the maximum deflection as a function of time. 

## 2. Development of CFRD Deformation Monitoring System Using FOG

### 2.1. Working Principle of the FOG

**Figure 1:**
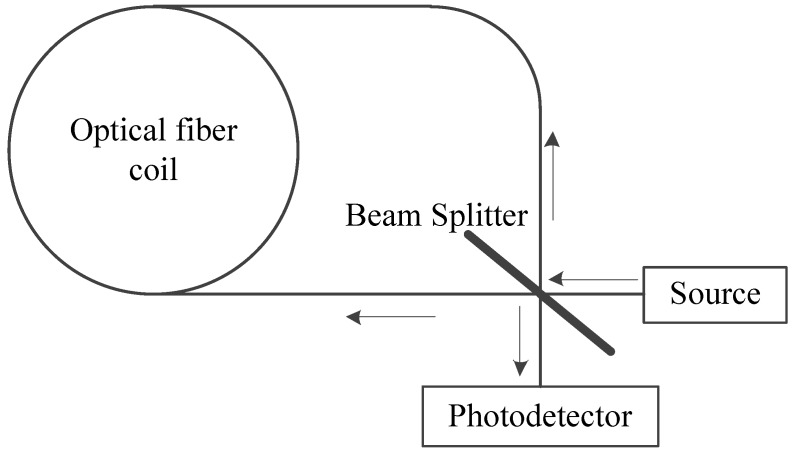
Working principle of FOG.

Fiber optic gyroscope (FOG) is a sensitive angular velocity sensor based on the Sagnac effect which was first discovered by Georges Sagnac in 1913. The basic concept of the Sagnac effect (Figure 1) is summarized as follows. A light source (S) generates a narrow spectral linewidth light beam. The beam later passes on an equal intensity Beam Splitter (BS), resulting in two light beams traveling around a closed ring fiber in opposite directions. After passing through the optical fiber coil, the two beams are superimposed again by the same beam splitter and guided to the photodetector (D). If the FOG is at rest, the two beams propagate the same distance, and thus, between them, no phase shift is observed. In contrast, as the FOG rotates about the normal vector on the fiber coil, the traveling distance of the two beams is different, and a phase difference appears. The relationship between the phase shift and FOG rotation rate is given as follows [20]:

(1) $$\Delta \varphi =\frac{4\pi RL}{\lambda c}\cdot \Omega$$

where Δφ is the phase shift, λ is the wavelength of the light source, *R* is the radius of fiber coil, L=2NπR is the total length of *N*-turn fiber, *c* is the speed of light, and Ω is the rotation rate of FOG.

### 2.2. Deformation Monitoring of CFRD Using FOG

This section presents the deformation monitoring system of CFRD using FOG and its working principle.

#### 2.2.1. Deformation Monitoring System

**Figure 2:**
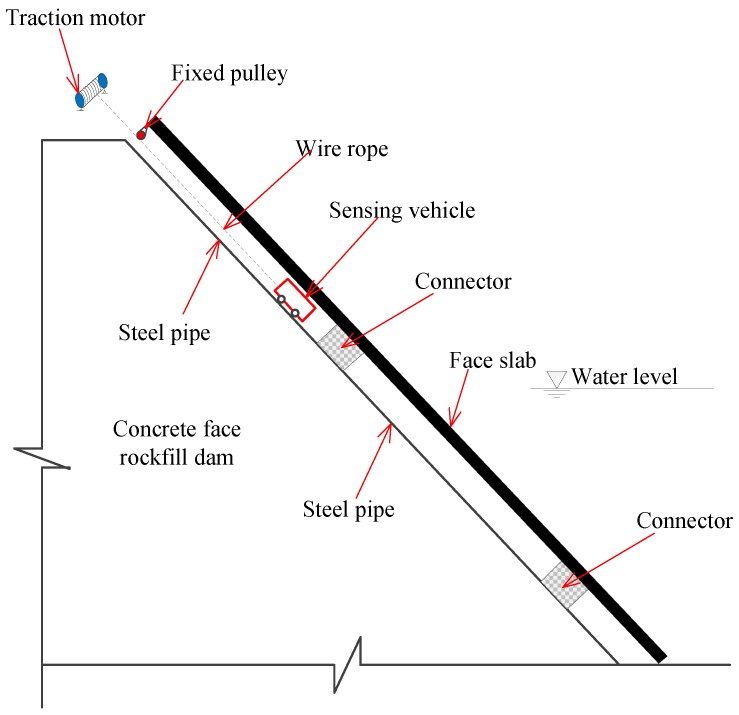
Deformation monitoring system of CFRD using FOG.

**Figure 3:**
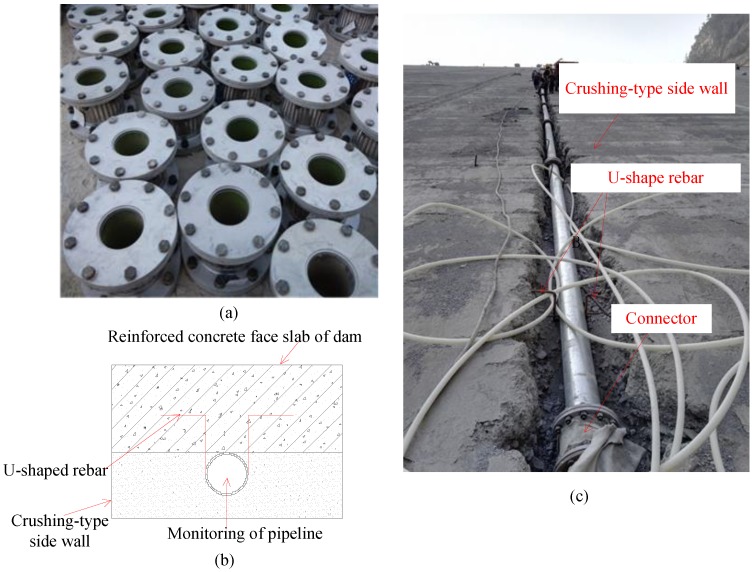
The monitoring pipeline: (**a**) Polyvinyl chloride polymer connectors; (**b**) A cross-section of monitoring pipeline; (**c**) In situ installation of monitoring pipeline.

Figure 2 shows the monitoring system for face slab deformation in CFRDs, which mainly consists of a monitoring pipeline and a sensing vehicle. The monitoring pipeline is made of seamless steel pipes with a length of 5 m, and polyvinyl chloride polymer connectors with a length of 0.3 m. As shown in Figure 3a, the connector is filled with six waves of double-layer polyurethane rubber and vulcanized at high temperature to increase its flexibility, smoothness, and durability. Both steel pipes and connectors have the same inner diameter (0.178 m) to ensure the smoothness of the monitoring pipeline. As shown in Figure 3b,c, the monitoring pipeline is embedded in a slot of the crushing-type side-wall beneath the concrete face slab, and its slope is the same as that of the face slab. A U-shaped rebar is used, with its U-shaped section welded to the pipeline and its two straight ends welded to reinforcement network of the reinforced concrete face slab. In this way, the monitoring pipeline and the face slab form a permanent monitoring channel during the construction stage which then serves to reflect deformations in the CFRD in service.

**Figure 4:**
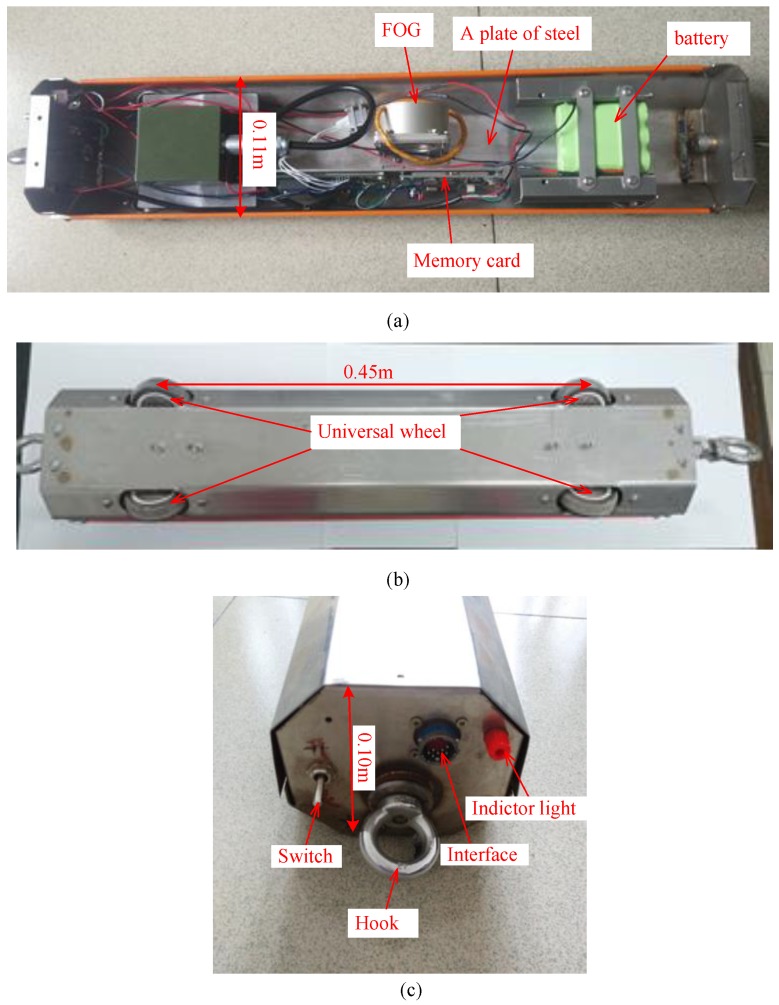
The sensing vehicle: (**a**) internal structure; (**b**) bottom view; (**c**) front view.

Figure 4 shows the structure of the sensing vehicle. The vehicle is 0.6 m long, 0.11 m wide, and 0.10 m high; its total weight is 8.5 kg. FOG is a core component of the sensing vehicle, which can sense changes in the vehicle’s angular velocity during the monitoring process. The data of FOG is stored in a memory card in real-time with a storage capacity of 8 GB. A 12V rechargeable battery is used as the power supply for the vehicle. A plate of steel (5.0 kg) is installed at the bottom of the sensing vehicle to increase its weight and to ensure that the vehicle can move close to the pipeline without turning over during the monitoring process. Four universal wheels are used to ensure the vehicle moves along a straight line (Figure 4b). The top panel of the vehicle is equipped with a switch, an indicator light, and a data transfer and charging interface (Figure 4c). The vehicle is controlled by a traction motor via a fixed pulley and wire robes.

#### 2.2.2. Working Principle

**Figure 5:**
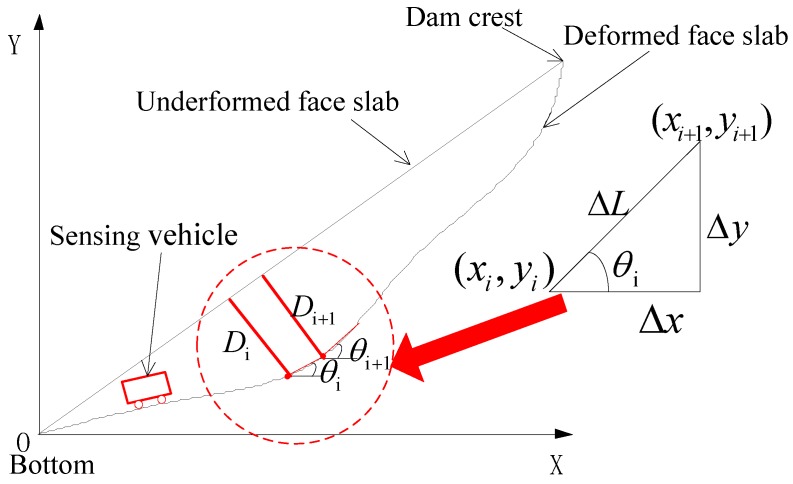
Working principle of dam deformation monitoring system.

Figure 5 presents the working principle of the CFRD deformation monitoring system under the action of water pressure. The sensing vehicle is driven by the traction motor and moves forward along the monitoring pipeline at a steady speed. The angular velocity of the FOG is recorded throughout the monitoring test, which is later used to calculate the deformation of the CFRD using quadratic integration method. A detailed derivation is given as follows.

As shown in Figure 5, the sensing vehicle reaches point (*x_i_*_+1_, *y_i_*_+1_) at time step (*i* + 1), and its position relates to the previous point (*x_i_*, *y_i_*) at time step *i* by the following equation:

(2) $$\begin{array}{c} x_{i+1}=x_{i}+\Delta x\\ y_{i+1}=y_{i}+\Delta y \end{array}$$

where Δx and Δy is the increment in the horizontal and vertical direction from point (*i*) to point (*i* + 1), respectively, as calculated by:

(3) $$\begin{array}{c} \Delta x=\Delta L\cdot \cos\theta_{i}=\Delta L\cdot \cos(\theta_{i-1}+\Delta \theta )\\ \Delta y=\Delta L\cdot \sin\theta_{i}=\Delta L\cdot \sin(\theta_{i-1}+\Delta \theta ) \end{array}$$

where *θ_i_* and *θ_i_*_+1_ are the *i*-th and (*i* ‒ 1)-th tangential angles of the deformation of CFRD, respectively; Δ*θ* and Δ*L* are the variations in the angle and distance between points *i* and (*i* ‒ 1), as expressed by:

(4) $$\begin{array}{c} \Delta \theta =\int_{t_{i-1}}^{t_{i}}\Omega dt\\ \Delta L=\int_{t_{i-1}}^{t_{i}}vdt \end{array}$$

Combining Equations (2)–(4), the position of the sensing vehicle at time step (*i* + 1) can be derived as follows:

(5) $$\begin{array}{c} x_{i+1}=x_{i}+\int_{t_{i-1}}^{t_{i}}vdt\cdot \;\cos(\theta_{i-1}+\int_{t_{i-1}}^{t_{i}}\Omega dt)\\ y_{i+1}=y_{i}+\int_{t_{i-1}}^{t_{i}}vdt\cdot \;\sin(\theta_{i-1}+\int_{t_{i-}{}_{1}}^{t_{i}}\Omega dt) \end{array}$$

Considering the fact that the sampling step is usually short and the vehicle is at a constant speed, Equation (5) can be simplified as follows:

(6) $$\begin{array}{c} x_{i+1}=x_{i}+v_{0}\cdot \Delta t\cdot \cos(\theta_{i-1}+\Omega \cdot \Delta t)\\ y_{i+1}=y_{i}+v_{0}\cdot \Delta t\cdot \sin(\theta_{i-1}+\Omega \cdot \Delta t) \end{array}$$

where v_0_ is the speed of the sensing vehicle and Δt is sampling interval of the FOG.

It is assumed that the slope ratio of the undeformed face slab of the dam is 1: *m*. Thus, the deflection of the face slab at point *i* is calculated as the perpendicular distance from the vehicle to the face slab:

(7) $$D_{j}=\frac{v_{0}\cdot \Delta t}{\sqrt{1+m^{2}}}\left(\sum_{i=1}^{i=j}\cos\theta_{i}-m\cdot \sum_{i=1}^{i=j}\sin\theta_{i}\right)$$

In this way, the deformation of CFRD is obtained and the deflection shape of the CFRD can be drawn. It is also worth mentioning that the recorded angular velocities of FOG are usually contaminated by noise that may arise through the roughness of steel pipes and connectors; a filter is therefore employed before the deformation calculation.

## 3. Implementation

**Figure 6:**
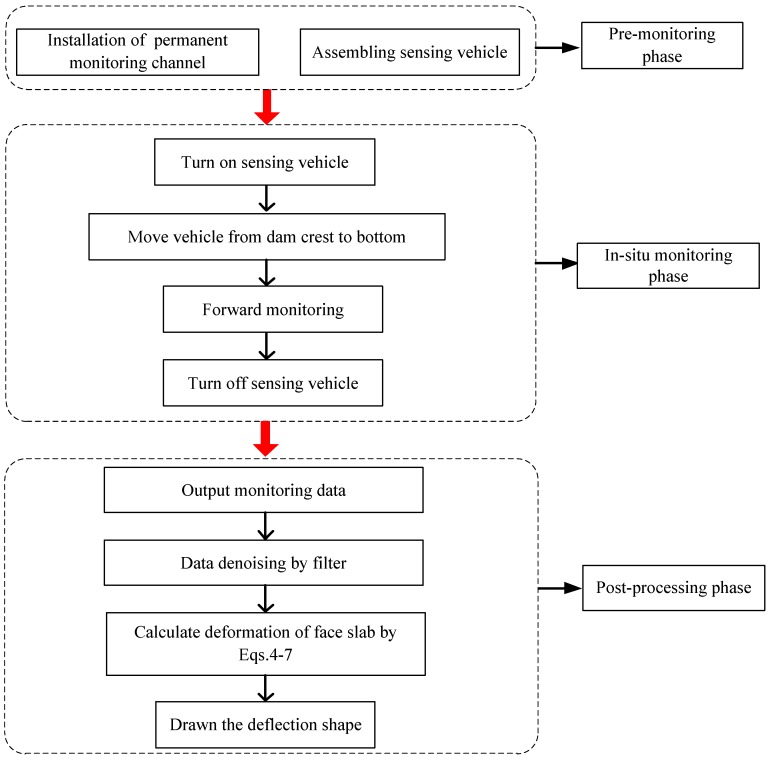
Flow chart of monitoring face slab deflection of CFRD using FOG.

Figure 6 shows a flow chart of the implementation of the deformation monitoring of CFRD using FOG, which consists of three phases: (a) a premonitoring phase, (b) an in situ monitoring phase, and (c) a postprocessing phase. In the premonitoring phase, a permanent monitoring channel is installed in a slot of the crushing-type sidewall in the construction stage, and a sensing vehicle is assembled with a FOG, memory card, 12V rechargeable battery, steel mass, and four universal wheels. In the in situ monitoring phase, the sensing vehicle was dropped down to dam bottom, stopped for 1 min, and dragged back from dam crest by controlling the traction motor; meanwhile, data from FOG was recorded and stored in the memory card. In the postprocessing phase, FOG data is filtered and the deformation shape of the CFRD is obtained.

## 4. In Situ Application of CFRD Deformation Monitoring System

### 4.1. Shuibuya CFRD

**Figure 7:**
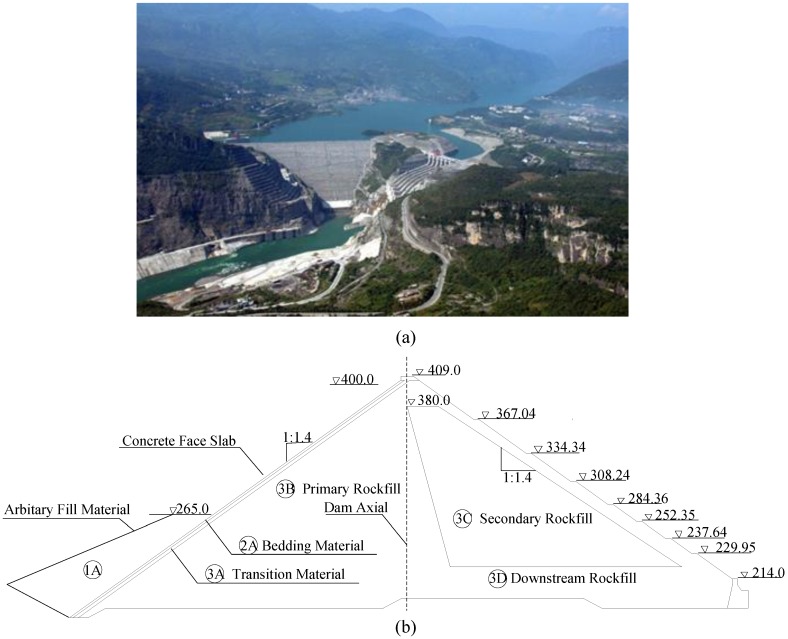
Overview of Shuibuya CFRD: (**a**) a general view of the Shuibuya CFRD; (**b**) a cross-section of the Shuibuya CFRD.

Shuibuya CFRD was constructed on the Qingjiang river in Badong county, in Enshi city, Hubei province, China (Figure 7a); it is 233 m tall, making it currently the tallest in the world. The construction project of Shuibuya CFRD was called the world CFRD landmark project by the International Commission on Large Dams. Figure 7b shows a typical cross-section of Shuibuya CFRD. The elevation of the dam crest is 409 m, and the normal water level of the reservoir is 400m. The upstream and downstream dam slopes are the same, i.e., equal 1:1.4. Construction on the dam began in October 2002 and was completed in July 2008, and it has been service since November 2008. The maximum reservoir capacity of the dam is approximately 4.59 × 10^9^ m^3^. It is equipped with four generators in the underground power plant with a total electricity generation capacity of 1840 MW.

### 4.2. In Situ Experimental Test

**Figure 8:**
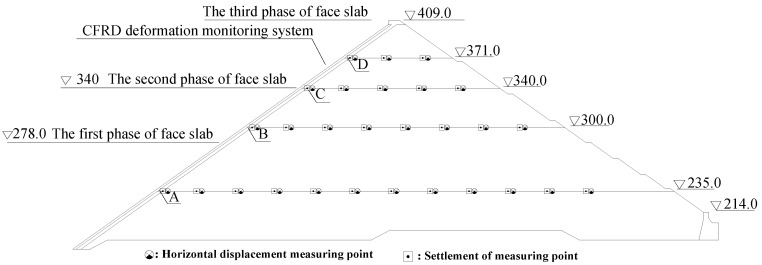
Layout of the in situ monitoring instrumentation at the maximum cross section (0+212).

Figure 8 shows a layout of the in situ instrumentation at the maximum cross-section (0+212) of Shuibuya CFRD, which consists of the present FOG-based CFRD deformation monitoring system, 39 hydraulic overflow settlement gauges (HOSGs), and 39 tension wire alignments (TWAs). The latter two sensors are to measure vertical settlement and horizontal displacements, respectively, among which the results near the face slab (Points A, B, C, and D) are used for validation of the present FOG-based CFRD deformation monitoring system. It is also worth mentioning that, except the aforementioned instrumentation, 45 traditional inclinometers were installed during the construction of the Shuibuya CFRD; however, all of them malfunctioned because their pipelines were crushed by the high water pressure.

**Figure 9:**
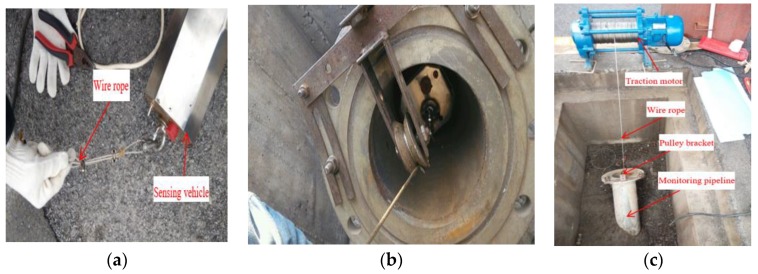
In situ monitoring of face slab deformations of Shuibuya CFRD: (**a**) the wire rope and the sensing vehicle; (**b**) the vehicle in the monitoring pipeline; (**c**) the working state of the system.

Figure 9 shows an experimental setup for the monitoring face slab deformation of the CFRD in December 2014. The in situ monitoring test was carried out following the in situ monitoring phase in Section 3. Figure 9a shows the sensing vehicle connected with the wire rope of the traction motor. Figure 9b shows the vehicle in the monitoring pipeline, in which a pulley bracket was installed at the pipeline orifice to prevent friction between the wire rope and the monitoring pipeline orifice, and to ensure that the sensing vehicle moved along a straight line in the pipeline. The sensing vehicle will steadily move from the dam crest to bottom, and then from the dam bottom to the crest by controlling the traction motor, as shown in Figure 9c. It takes about 40 min for one run of monitoring. The sampling frequency is 30 Hz. Two independent measurements were carried out for each test; the time interval between the two measurements was 20 min, to ensure the same initial temperature of the FOG.

### 4.3. Results and Discussion

#### 4.3.1. Data Processing

**Figure 10:**
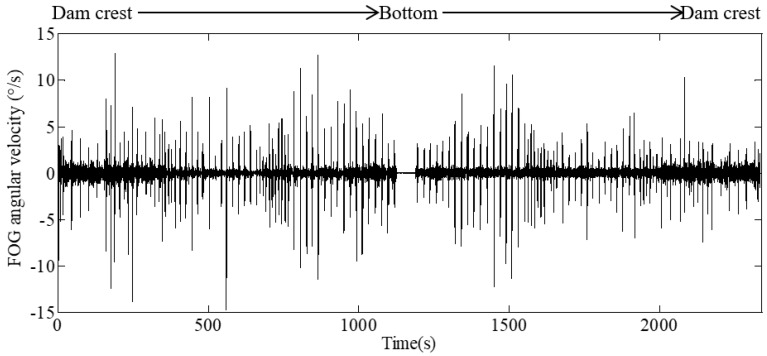
A typical set of angular velocity of CFRD monitoring system.

Figure 10 shows a typical set of angular velocities from the CFRD deformation monitoring system. Some of the data are about zero at the bottom of the dam, which indicates that the sensing vehicle stays at the bottom for one minute. It can also be seen that the angular velocities of the sensing vehicle moving from dam crest to bottom are almost symmetrical with those of the vehicle moving from dam bottom to crest. The data of the vehicle moving from dam bottom to crest is more stable and less fluctuant. The main reason for this is that extra angular velocities are generated because of the self-weight effects of the vehicle when it moves downward; in contrast, the vehicle will be pulled upward by the traction motor at a constant rate, and thus, it will move at a much steadier velocity. Therefore, the angular velocities of the sensing vehicle moving upward are selected as the effective measurement data in a complete monitoring process.

In the process of CFRD deformation monitoring with the FOG sensor, the sensing vehicle moves smoothly in the monitoring pipeline but vibrates considerably when it passes through the connection between the steel pipe and the connector. The vibration noise has a greater influence on the monitoring results and causes significant systematic errors. The major source of the noise is the loading frequencies related to the successive passing of the universal wheels of the sensing vehicle, which are a function of the length of the connector *L*_1_ = 30 cm, the distance between the front and rear universal wheels of the sensing vehicle *L*_2_ = 45 cm, and the velocity of the sensing vehicle *v* = 30 cm/s [21]. Specifically, the two loading frequencies are calculated by *f*_1_ = *v*/*L*_1_ =1.0Hz and *f*_2_ = *v*/*L*_2_ = 0.67Hz. Hence, a denoising filter needs to be used to eliminate vibration noise. In this paper, a low-pass filter was adopted for denoising with a threshold that was determined by a trial-and-error method. The performance of filter is quantified by the standard deviation (STD). Table 2 shows the STD results with three thresholds (2.0, 0.8, and 0.6 Hz). The first threshold (2 Hz) is higher than *f*_1_ and *f*_2_, the second (0.8 Hz) is higher than *f*_2_ but lower than *f*_1_, and the third (0.6 Hz) is lower than *f*_1_ and *f*_2_. It is observed that the filter with a threshold of 0.6 Hz has the minimum STD. Thus, a low-pass filter with a threshold of 0.6 Hz is hereafter used for denoising the measured angular velocities.

**Figure 11:**
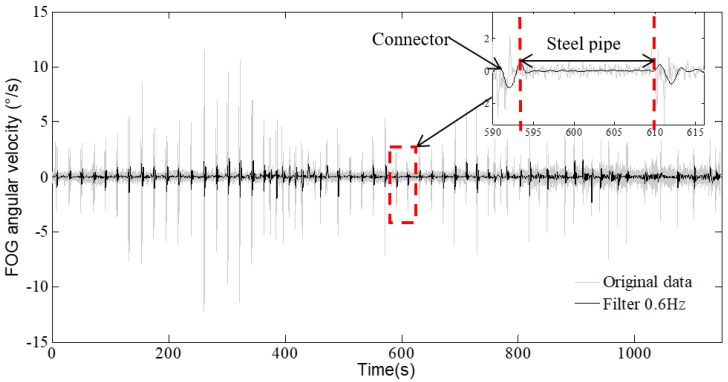
FOG angular velocity denoised by a low-pass filter.

Figure 11 shows the denoised angular velocities of the sensing vehicle using a low-pass filter with a threshold of 0.6 Hz. It is obvious that the noise of the angular velocities is greatly reduced and the bone curve of the measured angular velocities is retained. It can also be observed from the zoom-in section of the curve that the angular velocities vary near the connectors, and are rather smooth in the steel pipe. This conforms to the design principle of the monitoring pipeline, which states that the deformation of the pipeline mainly occurred at the connectors, while the steel pipe underwent little deformation. It takes about 17 s for the sensing vehicle to pass across one steel pipe. The speed of the sensing vehicle is about 30 cm/s; thus, the distance that the vehicle travels is about 5 m, which is equal to the length of a steel pipe in one cycle.

**Figure 12:**
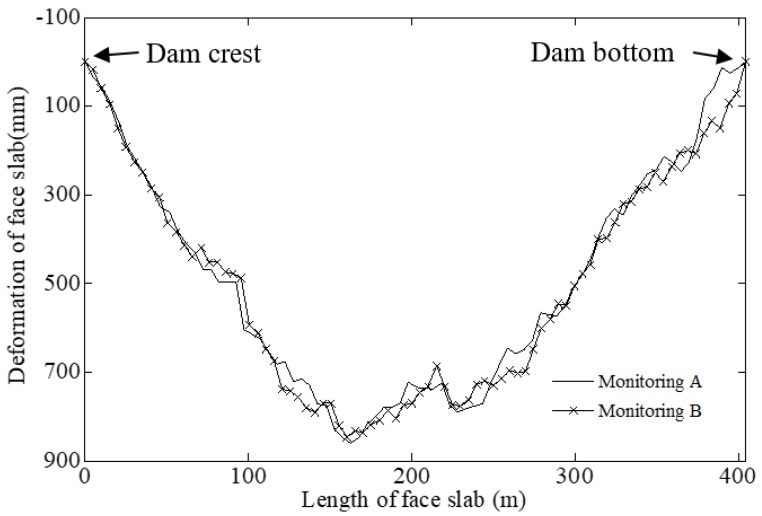
Two independent measurements of face slab deflection of Shuibuya CFRD.

The filtered angular velocities were substituted into Equations (4)–(7) in Section 2.2.2 to calculate the face slab deflection of CFRD. Figure 12 shows the face slab deflection in two independent measurements (Monitoring A and Monitoring B) at one in situ test. The deflection has a D-shape distribution, which is consistent with results in [13,22,23,24]. The deflection shapes in two measurements correspond very well with a correlation coefficient of 0.97. This confirms the excellent repeatability in two independent measurements, indicating that the performance of the novel FOG monitoring system is stable. The final results of the deflection are calculated by the average of two independent measurements.

#### 4.3.2. Test Results

**Figure 13:**
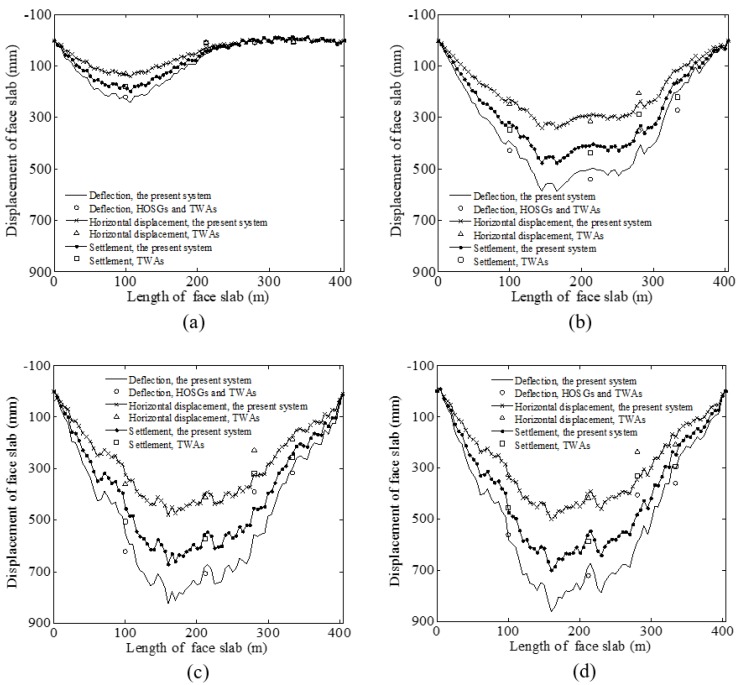
A comparison of the face slab deflection at the 0+212 cross section of Shuibuya CFRD between the novel FOG monitoring system and the traditional instruments: (**a**) measured on 24 April 2007; (**b**) measured on 21 November 2008; (**c**) measured on 10 August 2011; (**d**) measured on 17 June 2013. (HOSGs: hydraulic overflow settlement gauges, TWAs tension wire alignments).

Figure 13 shows the typical deflection of the face slab measured by the FOG monitoring system and traditional instruments in different years (2007, 2008, 2011, and 2013) when the water levels of the reservoir were 274 m, 399 m, 389 m, and 376 m, respectively. The reservoir started filling on 24 April 2007, and was full (at the water level of 400 m) on 21 November 2008. It can be seen from Figure 13a that the maximum deflection was about 246 mm, which occurred at a face slab length of 100 m (at about the elevation of 235 m); the deflection above 200 m tends to be zero. As the water level continued to increase to 399 m, the deflection shape of the face slab exhibited a D-shaped distribution, as shown in Figure 13b. Meanwhile, the maximum deflection was much larger (about 620 mm) and moved to a higher position of the face slab (at about the elevation of 278 m). As shown in Figure 13b,d, the maximum deflection increased from 2008 to 2013, although the water level of the reservoir in those years was stable. This time-dependent behavior was mainly attributed to the creep effect of the dam body made of coarse granular materials with strong permeability. Similar phenomena were also reported in the Miaojiaba [13] and Daegok dams [25]. The location of the maximum deflection in Figure 13b,d was at a slab face length of about 175 m. This is consistent with the observation in [24,26] that the maximum deflection of CFRD is at about one-third to one-half of the slab face.

To validate the effectiveness of the present FOG-based CFRD deformation monitoring system, the measured results at Points A, B, C, and D (in Figure 8) using HOSGs and TWAs were compared with those from the present system. As shown in Figure 13, the deflections of the face slab measured by the present system agreed quite well with those obtained from the four HOSGs and TWAs, regardless of the water level. The latter was calculated by $d_{t}=\sqrt{d_{vt}^{2}+d_{ht}^{2}}$ with *d*_t_, *d*_vt_, and *d*_ht_ being defined as the deflection of the face slab calculated from data of HOSGs and TWAs, the measured vertical settlement from HOSGs, and the measured horizontal displacement from TWAs, respectively. To further analyze the deformation characteristics of the face slab, the deflection of the face slab measured using the present system (*d*_p_) is decomposed into the vertical settlement term (*d*_vp_) and the horizontal displacement term (*d*_hp_). It can be found in Figure 13 that the agreement still holds between the decomposed vertical settlement term and the results of HOSGs, and between the horizontal displacement term and the results of TWAs. Meanwhile, it was also observed that the vertical settlement term contributed about 80% to the face slab deflection, and that the horizontal displacement term has relatively less contribution. In other words, the main factor causing the face slab deflection is the vertical settlement of the dam body, probably, due to the fact that granular materials become denser under pressure.

**Figure 14:**
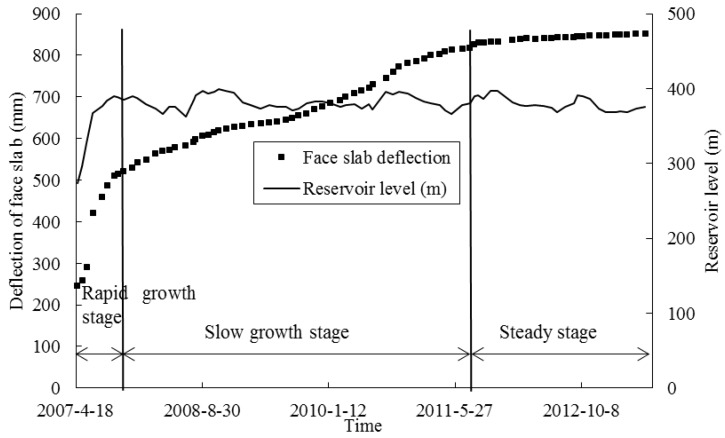
Measured maximum deflection during the reservoir filling and after five years.

Figure 14 shows the variation of the measured maximum deflection by the FOG monitoring system during the reservoir filling and in the following five years. In the figure, the water level as a function of time is also superimposed. The deformation of the face slab of the dam can be broadly categorized into three stages: (I) the rapid growth stage during the filling of reservoir (April 2007~November 2007), (II) the slow growth stage in the first three years after the end of filling (November 2007~August 2011), and (III) the steady stage until June 2013. In Stage I, the deflection of the face slab rapidly increased under the action of water pressure during the filling of reservoir. In this stage, the maximum incremental deflection was as much as 374 mm, indicating that the face slab deformation was significantly affected by water pressure. In Stage II, the face slab deflection increased much slowly, and the deformation rate was approximately 5 mm per month. In Stage III, the deformation of the face slab tended to be stable, and the deformation rate was approximately 1 mm per month. On average, the deformation rate of the Shuibuya CFRD was 3.4 mm in the latter two stages. This is consistent with the conclusion in [27] that the average deformation rate of the face slab is about 3 mm per month during the first ten years after the end of filling. The continuous growth of the maximum deflection of the dam in these two stages is because of, as mentioned, the creep effect of coarse granular materials in the dam body, although the water level was kept at an average level of around 390 m. 

#### 4.3.3. Discussion

In this paper, the proposed FOG monitoring system was designed to be applied for deformation monitoring of the face slab of a CFRD. In this application, the deformation of the CFRD usually ranged from 1 mm to a few meters. Thus, it requires that the FOG monitoring system have a large dynamic measurement range with satisfactory accuracy. The monitoring pipeline of the FOG monitoring system must be consistent with the deformation of face slab, and should be able to provide long-term service for dam deformation monitoring. Meanwhile, the monitoring system needs to be resistant to harsh environments, such as high water pressures and humidity levels, temperature variations, and vibrations. Therefore, the proposed FOG monitoring system in this study needs to overcome the above difficulties. The proposed monitoring system has the following advantages:

(a) The monitoring pipeline of the FOG monitoring system is installed with little interference to the dam construction, which is connected with the steel mesh of the concrete face slab without special protection. The monitoring pipeline forms a permanent monitoring channel under the construction stage, which can provide long-term and effective monitoring for the safe operation of the dam. In the Shuibuya CFRD, all the traditional inclinometers malfunctioned because of the excessive water pressure; however, the FOG monitoring system has been in service since the start of reservoir filling. It is worth mentioning that the FOG sensors can be upgraded as long as the monitoring pipeline works.
(b) The FOG monitoring system is a distributed measurement that provides more monitoring information than the point measurement of traditional instruments. When the sensing vehicle runs in the monitoring pipeline, the angular velocity in a monitoring process can be collected, and the deformation of the whole monitoring section face slab is obtained.
(c) The proposed FOG monitoring system can easily and automatically measure the face slab deformation of CFRD. During the monitoring process, the sensing vehicle is connected to the motor by a wire rope and then placed into the monitoring pipeline, which automatically collects the angular velocity.

Although the proposed FOG monitoring system has many such advantages, it still needs to be improved in many aspects. For example, the precision of the FOG monitoring system is limited by the long-term drift of the FOG. Furthermore, the measured angular velocities are greatly contaminated by multiple impacts due to the successive passage of the sensing vehicle on the connector. In this paper, a low pass filter was designed and employed for denoising, and the results were fairly acceptable. In order to further improve the denoising capability, more advanced denoising methods, such as Wavelet transforms, moving average (MA), empirical mode decomposition (EMD), Kalman filter (KF), and its adaptive variant, can be adopted. The monitoring pipeline is connected by steel pipes and connectors. In the future, it will be necessary to use polymer materials as the monitoring pipeline, which can not only reflect the deformation of the dam more realistically, but also reduce the engineering costs. At present, the monitoring process requires manual operation, and further research can be conducted on remote, automatic, intelligent monitoring.

## 5. Conclusions

A FOG-based deformation monitoring system was designed, developed, and analyzed in this study to monitor the face slab deformation of a CFRD. The system consisted of the permanent monitoring pipeline and the sensing vehicle. The working principle of the deformation monitoring system was deduced. A low-pass filter was designed to eliminate the vibration noise of the angular velocities of the sensing vehicle during the monitoring process. For each test, two independent measurements were conducted; their correlation coefficient was 0.97, confirming the repeatability of the system. The FOG monitoring system was validated through a comparison with traditional instrument measurements. In situ test results of Shuibuya CFRD in the first six years of service showed that the deflection of the face slab was D-shaped, and that it could be broadly categorized into the rapid growth stage during the filling of reservoir (April 2007~November 2008), the slow growth stage in the first three years after the end of filling (November 2008~August 2011), and the steady stage up to June 2013.

## Figures and Tables

**Table 1:** Representative CFRDs higher than 200 m.

No	Name	Location	Height, H (m)	Length, L (m)	Construction Situation
1	Campos Novos	Brazil	202	590	Completed in 2006
2	Shuibuya	China	233	660	Completed in 2008
3	Bakun	Malaysia	203.5	750	Completed in 2009
4	Houziyan	China	223.5	283	Completed in 2016
5	Jiangpinghe	China	219	414	under construction
6	La Yesca	Philippines	205	629	under construction
7	Nam Ngum 3	Laos	220	-	planned
8	Morro de Arica	Peru	221	-	planned
9	Agbulu	Philippines	234	-	planned
10	Gushui	China	242	430	planned
11	Dashixia	China	251	598	planned
12	Cihaxia	China	253	669	planned
13	Rumei	China	315	-	planned

**Table 2:** STD results for different low-pass filter thresholds.

	Original	2HZ	0.8HZ	0.6HZ
STD	1.076	0.728	0.423	0.281
